# Assessing sub-regional-specific strengths of healthcare systems associated with COVID-19 prevalence, deaths and recoveries in Africa

**DOI:** 10.1371/journal.pone.0247274

**Published:** 2021-03-01

**Authors:** Iddrisu Amadu, Bright Opoku Ahinkorah, Abdul-Rahaman Afitiri, Abdul-Aziz Seidu, Edward Kwabena Ameyaw, John Elvis Hagan, Eric Duku, Simon Appah Aram

**Affiliations:** 1 Africa Centre of Excellence in Coastal Resilience, University of Cape Coast, Cape Coast, Ghana; 2 Department of Fisheries and Aquatic Sciences, School of Biological Sciences, College of Agriculture and Natural Sciences, University of Cape Coast, Cape Coast, Ghana; 3 School of Public Health, Faculty of Health, University of Technology Sydney, Ultimo, New South Wales, Australia; 4 Department of Environmental Science, School of Biological Sciences, College of Agriculture and Natural Sciences, University of Cape Coast, Cape Coast, Ghana; 5 Department of Population and Health, College of Humanities and Legal Studies, University of Cape Coast, Cape Coast, Ghana; 6 College of Public Health, Medical and Veterinary Sciences, James Cook University, Townsville, Queensland, Australia; 7 Department of Health, Physical Education, and Recreation, University of Cape Coast, Cape Coast, Ghana; 8 Neurocognition and Action-Biomechanics-Research Group, Faculty of Psychology and Sport Sciences, Bielefeld University, Bielefeld, Germany; 9 College of Safety and Emergency Management Engineering, Taiyuan University of Technology, Taiyuan, Peoples Republic of China; National Institute for Infectious Diseases Lazzaro Spallanzani-IRCCS, ITALY

## Abstract

**Introduction:**

The coronavirus 2019 (COVID-19) has overwhelmed the health systems of several countries, particularly those within the African region. Notwithstanding, the relationship between health systems and the magnitude of COVID-19 in African countries have not received research attention. In this study, we investigated the relationship between the pervasiveness of the pandemic across African countries and their Global Health Security Index (GHSI) scores.

**Materials and methods:**

The study included 54 countries in five regions viz Western (16); Eastern (18); Middle (8); Northern (7); and Southern (5) Africa. The outcome variables in this study were the total confirmed COVID-19 cases (per million); total recoveries (per million); and the total deaths (per million). The data were subjected to Spearman’s rank-order (Spearman’s rho) correlation to determine the monotonic relationship between each of the predictor variables and the outcome variables. The predictor variables that showed a monotonic relationship with the outcome were used to predict respective outcome variables using multiple regressions. The statistical analysis was conducted at a significance level of 0.05.

**Results:**

Our results indicate that total number of COVID-19 cases (per million) has strong correlations (*r*_*s*_ >0.5) with the median age; aged 65 older; aged 70 older; GDP per capita; number of hospital beds per thousand; Human Development Index (HDI); recoveries (per million); and the overall risk environment of a country. All these factors including the country’s commitments to improving national capacity were related to the total number of deaths (per million). Also, strong correlations existed between the total recoveries (per million) and the total number of positive cases; total deaths (per million); median age; aged 70 older; GDP per capita; the number of hospital beds (per thousand); and HDI. The fitted regression models showed strong predictive powers (R-squared>99%) of the variances in the total number of COVID-19 cases (per million); total number of deaths (per million); and the total recoveries (per million).

**Conclusions:**

The findings from this study suggest that patient-level characteristics such as ageing population (i.e., 65^+^), poverty, underlying co-morbidities–cardiovascular disease (e.g., hypertension), and diabetes through unhealthy behaviours like smoking as well as hospital care (i.e., beds per thousand) can help explain COVID-19 confirmed cases and mortality rates in Africa. Aside from these, other determinants (e.g., population density, the ability of detection, prevention and control) also affect COVID-19 prevalence, deaths and recoveries within African countries and sub-regions.

## Introduction

The coronavirus 2019 (COVID-19) has overwhelmed the health systems of several countries, particularly those within the African region [1, 2]. The Global Health Security Index (GHSI), which happens to be the first assessment of countries’ readiness to overcome outbreaks such as COVID-19 was released in 2019. The outcome of the initial evaluation with the index indicated that no country among the 195 countries assessed was sufficiently prepared for pandemic or disease outbreaks, thus indicating critical shortfall in pandemic or outbreak preparedness [3]. The index consists of 34 indicators, 85 sub-indicators and 6 categories.

To overcome COVID-19, the World Health Organisation (WHO) has provided interim guidelines for the global community [4]. Similarly, the Center for Disease Control (CDC) has advocated the implementation of key strategies at the community level. These comprise emergency plans, contact tracing and case identification [5]. The Scientific and Technical Advisory Group for Infectious Hazards of the WHO has also outlined at least nine principal strategies needed for the containment and elimination of the pandemic [6]. Recommendations such as self-quarantine, social distancing and several personal hygiene measures have also been suggested [7].

Robustness of health systems at the regional and national levels cannot be disentangled from how well these recommendations can be implemented. Further, a robust and well-planned health system with adequate essential resources such as oxygen, ventilators and Personal Protective Equipment (PPE) is required to provide the needed COVID-19 curative and preventive care [8]. Conversely, sub-standard health system translates to low-quality healthcare with associated complications [9]. Thus, sub-standard care reflects in unsafe or inadequate clinical facilities or practices, medication errors, wrong diagnosis inter alia [9].

COVID-19 has exposed weak health systems globally thereby necessitating health system strengthening [10]. Even before the pandemic, Africa’s health system had been identified to be ailing with several challenges emerging from insufficient budgetary allocation to health, relatively bad leadership and management as well as inadequate health workforce [11–13]. Past epidemics within the African region such as Ebola in Central Africa and Lassa fever in Nigeria have further weakened health systems of the region [13]. For instance, the Ebola outbreak in Sierra Leone led to 23 per cent reduction in healthcare delivery, with associated 4,000 extra stillbirths, neonatal and maternal deaths, as well as about 2,800 additional TB, HIV and malaria deaths [14].

As a result, even in the event of a flattened curve scenario of COVID-19, health systems in Africa could be overwhelmed [14]. Due to these and other factors, it is not surprising that Africa often experiences the most dramatic impacts of public health crises, with the WHO forecasting that the COVID-19 pandemic could disproportionately affect Africa [15]. In Africa, first countries to have confirmed COVID-19 cases such as Egypt and Algeria are not the countries with the highest cases presently [16]. By 20^th^ October 2020, Africa had 1,262,476 cases with 28,601 deaths. South Africa alone accounted for 56% (706,304) of all confirmed cases followed by Ethiopia (90,490 cases; 7.2%) and Nigeria (61,630 cases; 4.9%). Coincidentally, other African countries are recording as low as 148 (Seychelles; 0.01%), 421 (Mauritius; 0.03%) and 452 (Eritrea; 0.04%) cases. COVID-19-induced deaths have also varied substantially across African countries and ranges from 65% (i.e. 18 656) in South Africa to 0.0% (i.e. 0) in Seychelles and Eritrea [17]. Could these variations reflect the robustness of health systems across African countries? Although this question is unanswered, established relationship and hypotheses between health systems’ efficacy and health outcomes [9] unquestionably suggest that the pattern and variation in COVID-19 prevalence and fatality across Africa cannot be comprehended without contextualizing the pandemic within the strengths of the health system of African countries.

The relationship between health systems as well as other theoretically relevant variables and magnitude of COVID-19 in African countries have not received research attention, despite the aforementioned. Previous COVID-19 studies have prioritised the need to overcome fake news about the pandemic [18], the importance of testing [19] as well as the pervasiveness of the pandemic in Africa and the continent’s response [20]. This proposed study seeks to advance frontiers of COVID-19 knowledge by investigating the relationship between the pervasiveness of the pandemic across African countries and their GHSI scores, determine the order and magnitude of predictor variables on confirmed positive, recovered and death cases.

## Materials and methods

### Study countries

**Fig 1:**
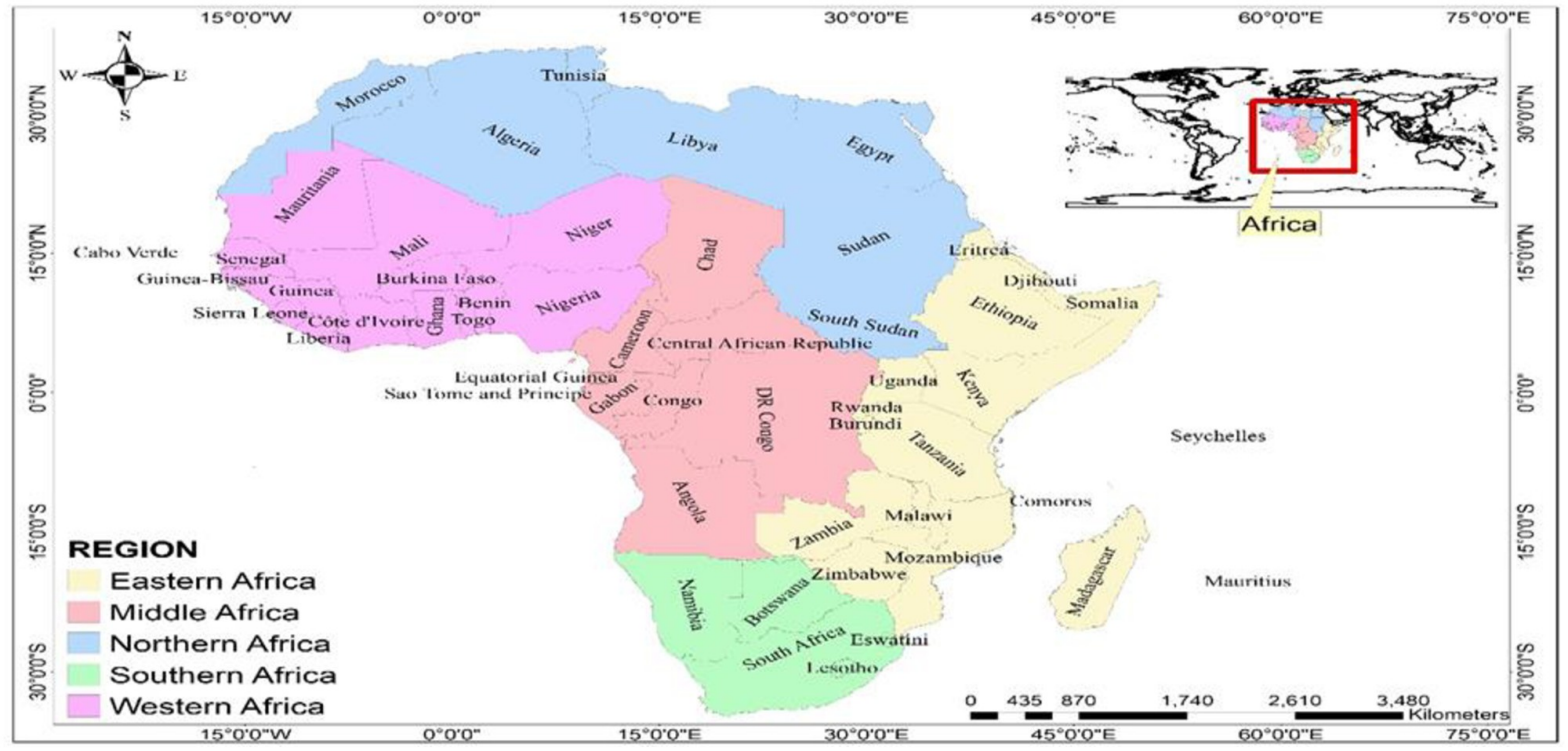
Map of study countries (reprinted from https://tapiquen-sig.jimdofree.com/descargas-gratuitas/mundo/, under a CC BY license, with permission from Carlos Efrain Porto Tapiquen, 2021).

The study included 54 countries in 5 regions viz Western (16); Eastern (18); Middle (8); Northern (7); and Southern (5) of Africa (Fig 1). These countries were assessed for data availability before inclusion in the study. The criteria for inclusion were (i) the availability of nationally representative data on COVID-19; and (ii) Global Health Security Index (GHSI) parameter scores.

### Data sources

The data on COVID-19 and other relevant variables were collected from the COVID-19 database by Our World in Data [21], and that of the Johns Hopkins University Center for Systems Science and Engineering (JHU CCSE). Data on variables including total cases (per million); total deaths (per million); population density; median age; aged 65 older; aged 70 older; GDP per capita; extreme poverty; cardiovascular death rate; diabetes prevalence; female smokers; male smokers; handwashing facilities; hospital beds per thousand; life expectancy; and Human Development Index (HDI) as at 5^th^ November 2020 were extracted. Also, data on the total number of recoveries for each study country on 5^th^ November 2020 were extracted from the JHU CCSE COVID-19 dataset [22].

Data on the Global Health Security Index (GHSI) parameter scores were obtained from https://www.ghsindex.org/. For the first time, the Global Health Security Index presents data from a comprehensive country-level assessment and metrics of health security and strength of healthcare systems. The report covering a total of 195 countries was released in 2019. It includes country scores on parameters including overall GHSI score; capacity in terms of early detection and reporting of epidemics of potential international concern; ability to prevent the emergence or release of pathogens; rapid response to and mitigation of the spread of an epidemic; sufficient and robust health system to treat the sick and protect health workers; commitments to improving national capacity, financing and adherence to norms; the overall risk environment and the vulnerability of the country to biological threats.

Map data (shapefiles) for the study countries were obtained from Carlos Efraín Porto Tapiquén. Geografía, SIG y Cartografía Digital. Valencia, Spain, 2020 (http://tapiquen-sig.jimdofree.com) which has openly available map data.

### Outcome variables

**Fig 2:**
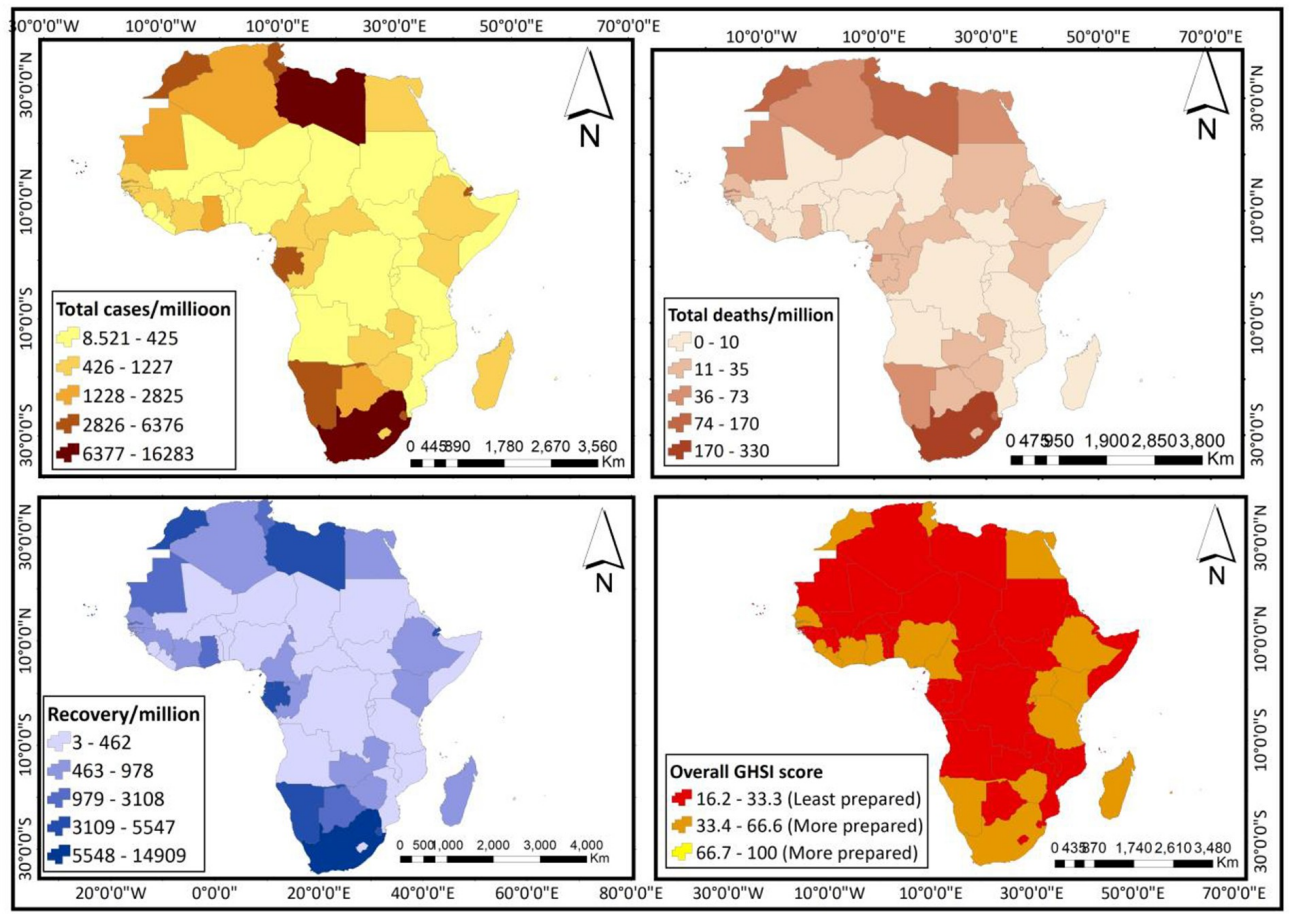
Spatial representation of data on outcome and key predictor variables (reprinted from https://tapiquen-sig.jimdofree.com/descargas-gratuitas/mundo/, under a CC BY license, with permission from Carlos Efrain Porto Tapiquen, 2021).

The outcome variables in this study included the total COVID-19 cases (per million); the total number of recoveries (per million); and the deaths recorded (per million). Data for these variables are on a discrete-continuous scale. These variables together with the key predictor (GHSI score) are spatially represented in Fig 2.

### Predictor variables

These variables included population density; median age; aged 65 older; aged 70 older; GDP per capita; extreme poverty; cardiovascular death rate; diabetes prevalence; female smokers; male smokers; handwashing facilities; hospital beds per thousand; life expectancy; and Human Development Index (HDI); and the GHSI parameters. All seven GHSI parameters namely, overall GHSI labelled “Overall GHS Index”; capacity to prevent the emergence or release of pathogens “Prevent”; capacity to rapidly respond to and mitigate the spread of an epidemic “Rapid response”; ability to detect and report epidemics of potential international concern early enough “Detection & Reporting”; the presence of a sufficient and robust health system not only capable of handling the sick but also protecting health workers “Sufficient & Robust HS”; commitments to improving national capacity, financing and adherence to norms “Commitments”; and overall risk environment and country vulnerability to biological threats “Overall risk environment” are scores between 0 and 100. For brevity, we used “variables” to include parameters.

### Data analyses

The data were analyzed using Stata 14 MP (StataCorp, College Station, TX, USA) software, and Microsoft Excel 2016. To understand the data relevant dynamics to this study, we performed descriptive statistics (frequencies, percentages and graphical representations). The relationship between the predictor variables and the outcome variables was examined using inferential statistics. We further incorporated the data into a GIS environment to produce relevant maps for easy visualization and to enhance understanding.

### Univariate analyses

Spearman’s rank-order (Spearman’s rho) correlation was first used to determine the monotonic relationship between each of the predictor variables and the outcome variables. The calculation of a correlation coefficient, *r*_*s*_ helps to inform the strength of association between two random variables [23, 24]. The correlation coefficient *r*_*s*_ ranges between -1 and 1, where a value closer to 1 signifies a strong positive correlation between x and y, both values increase or decrease together. A value closer to -1 means a strong negative association and thus, the value of y decreases as x increases. A coefficient, *r*_*s*_ closer to zero means the poorer the interrelationship. For effect size between variables, a correlation value of 0.1 means a weak association, 0.3 means a medium association and 0.5 means a high or strong association between the two variables [24]. The predictor variables that showed a monotonic relationship with the outcome variables at a statistical significance of 0.05 were used to predict respective outcome variables using multiple linear regression.

### Multivariable analysis

To understand the combined impact of the predictor variables on the outcome variables, we subjected the data to multivariable linear regression. A statistical significance level of 0.05 was set for all analyses.

## Results

### Univariate analyses results

**Fig 3:**
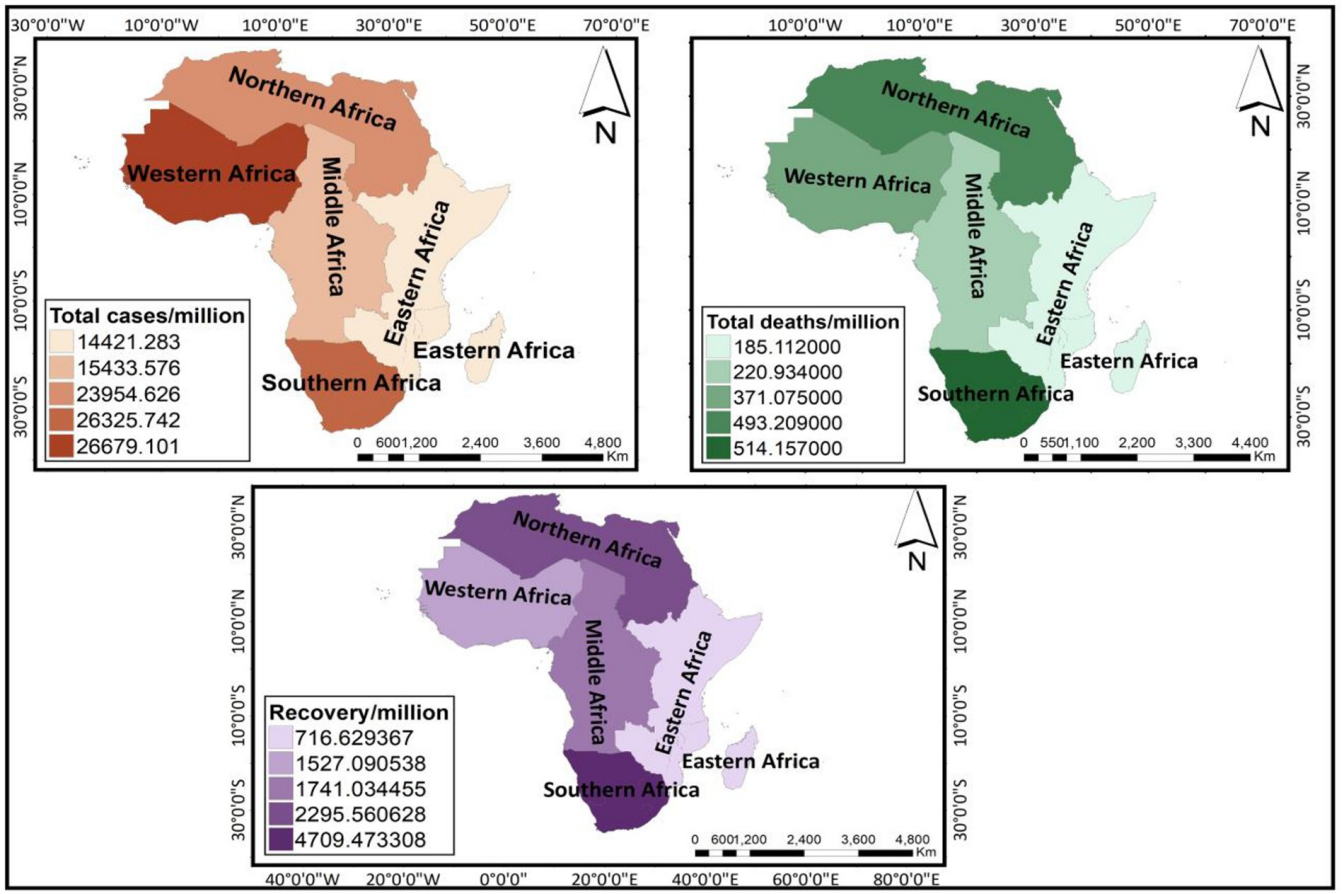
Maps showing confirmed positive cases, deaths and recoveries by regions of Africa (reprinted from https://tapiquen-sig.jimdofree.com/descargas-gratuitas/mundo/, under a CC BY license, with permission from Carlos Efrain Porto Tapiquen, 2021).

**Fig 4:**
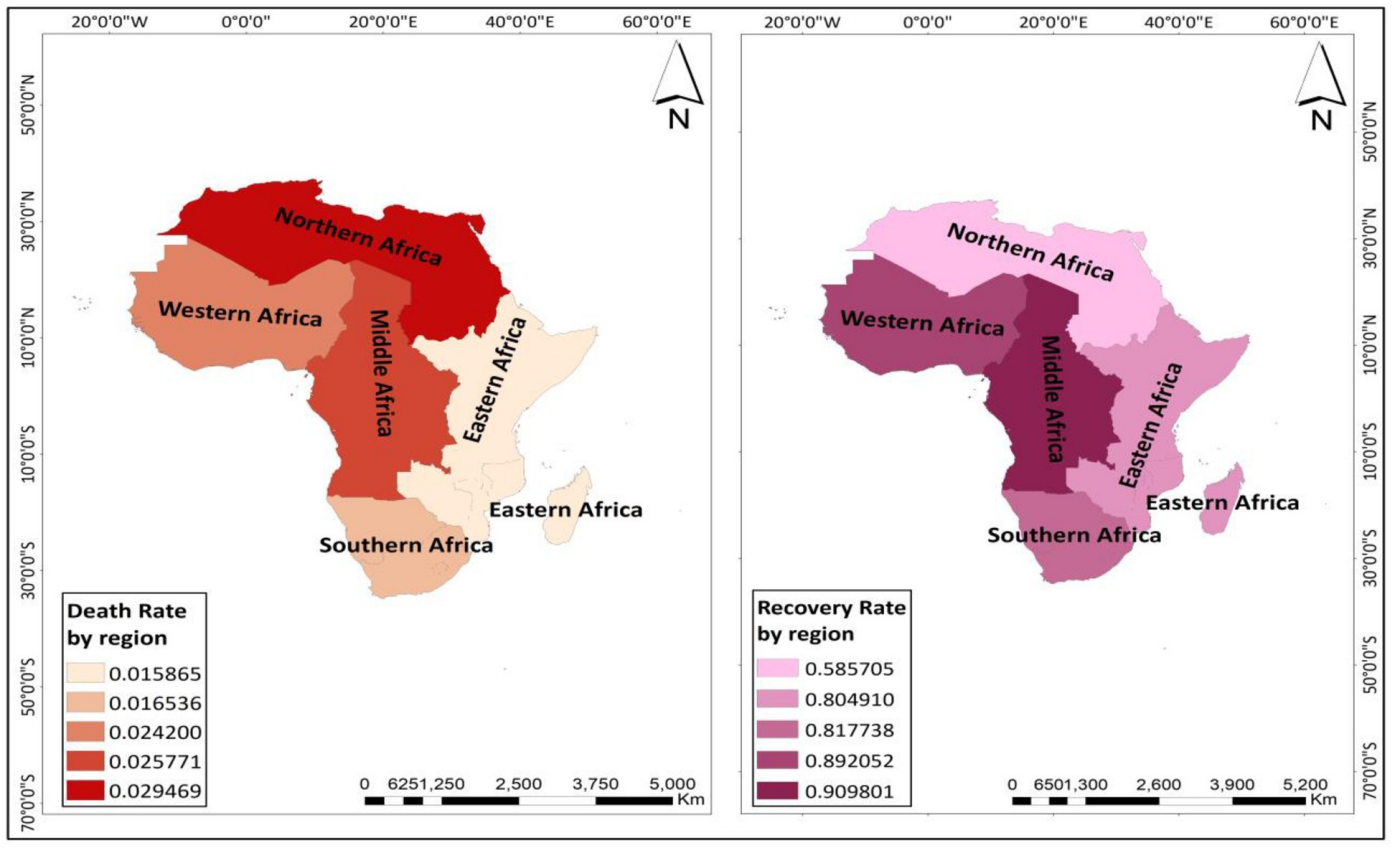
Maps showing the death rate (left) and recovery rate (right) by region of Africa (reprinted from https://tapiquen-sig.jimdofree.com/descargas-gratuitas/mundo/, under a CC BY license, with permission from Carlos Efrain Porto Tapiquen, 2021).

Descriptive statistics performed indicate that Western Africa had the highest number of confirmed COVID-19 positive cases (per million) (26,679) while the lowest was recorded in Eastern Africa (14,421). The highest and least numbers of COVID-19 deaths were also recorded in Southern Africa (514) and Eastern Africa (185) respectively. Averagely, the number of recoveries (per million) was highest in Southern Africa (4,709) whiles Eastern Africa showed the least number of recoveries (per million) (717). These are spatially represented in Fig 3. Northern Africa had the highest mean (0.03) COVID-19 death rate (deaths (per million)/confirmed positive cases (per million)) as indicated by the dark red colour on the map (left) in Fig 4. Eastern Africa recorded the lowest death rate (0.016). The recovery rate by region was also calculated as the number of recovered cases (per million) divided by the number of confirmed positive cases (per million). Here, considering that regions have an unequal number of countries, we divided the recovery rate of each region by its corresponding number of countries included in the study to ascertain the average recovery rate. The results showed that Middle Africa had the highest average recovery rate (0.91). This was followed by Western Africa (0.89), with Northern Africa recording the least average recovery rate (0.59). Fig 4 shows a spatial representation of death rates and average recovery rates by region of Africa.

Using the GHSI categorization (GHSI score 0 to = >33.3 as "Least prepared"; GHSI scores 33.4 to = >66.6 as "More prepared", and GHSI score 66.7 to = >100 as “Most prepared”), we calculated the number of confirmed positive cases, deaths and recovery for each category. All countries in Africa were either least prepared or more prepared.

**Fig 5:**
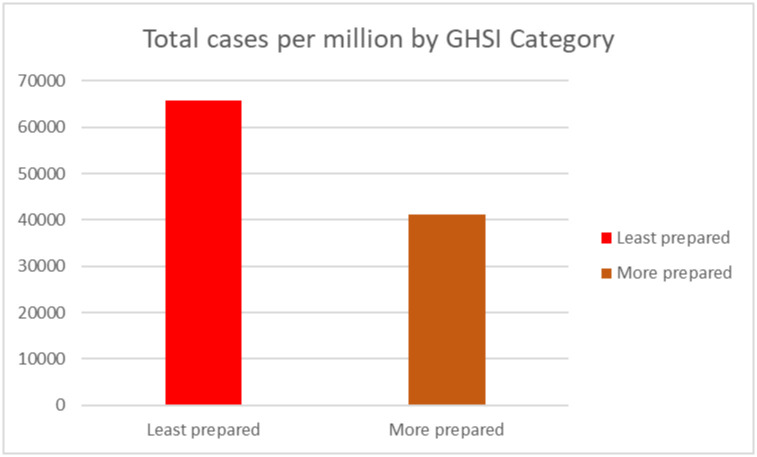
Total confirmed positive cases by GHSI category.

**Fig 6:**
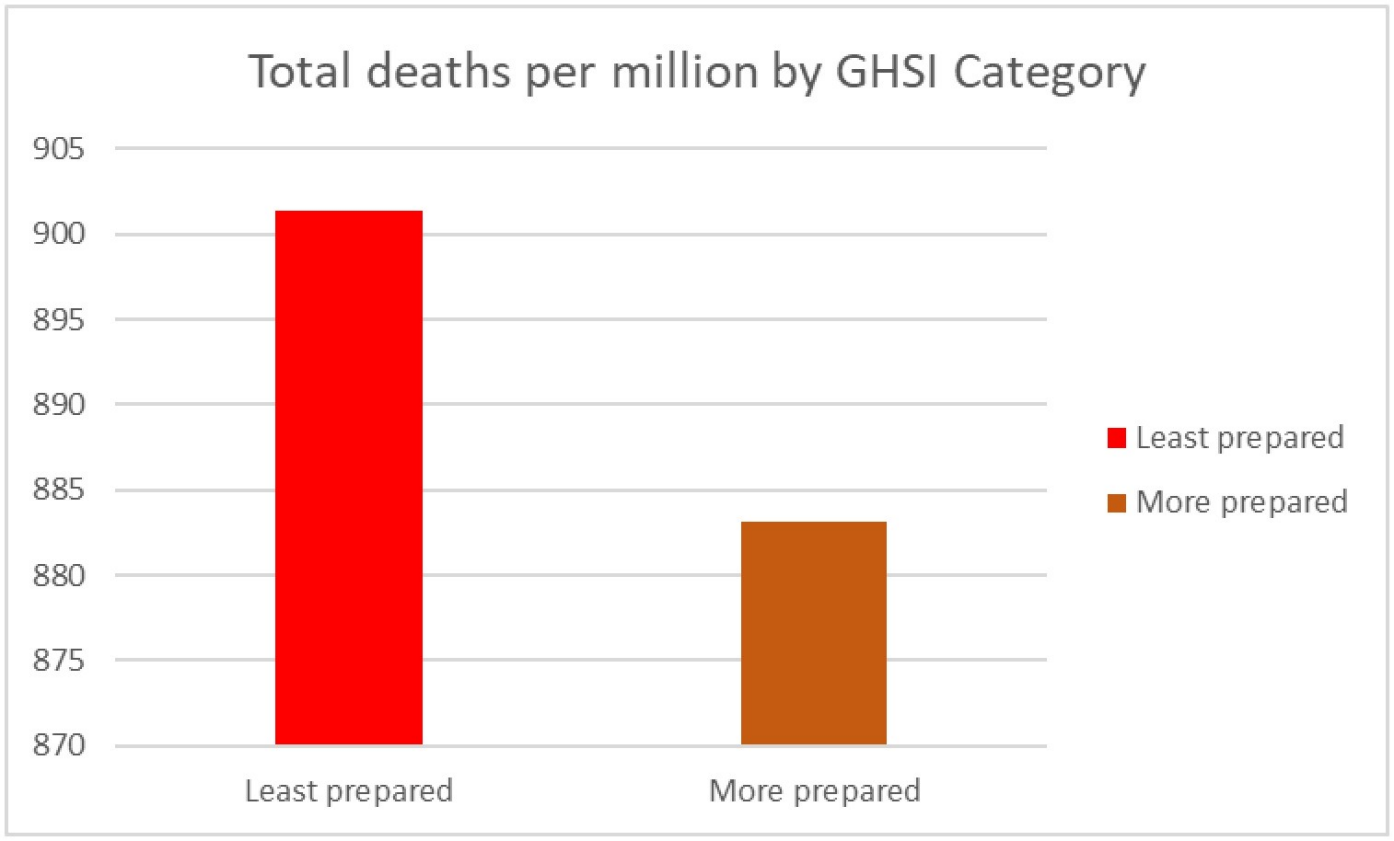
Total deaths by GHSI category.

**Fig 7:**
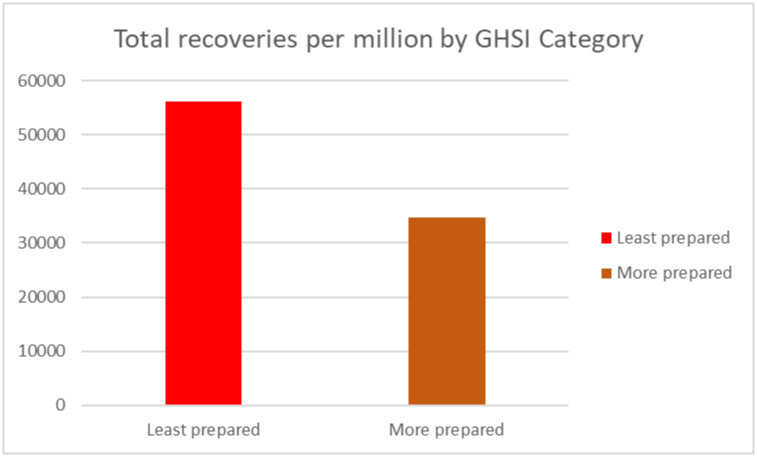
Total recoveries by GHSI category.

Worth noting, the total number of cases (per million) population and deaths (per million) of the population in “least prepared” countries for pandemics based on the GHSI categorization were high as compared to more prepared countries (see Figs 5 and 6). Surprisingly, the number of recoveries (per million) of population were high in the least prepared countries as compared to more prepared countries as shown in Fig 7.

**Fig 8:**
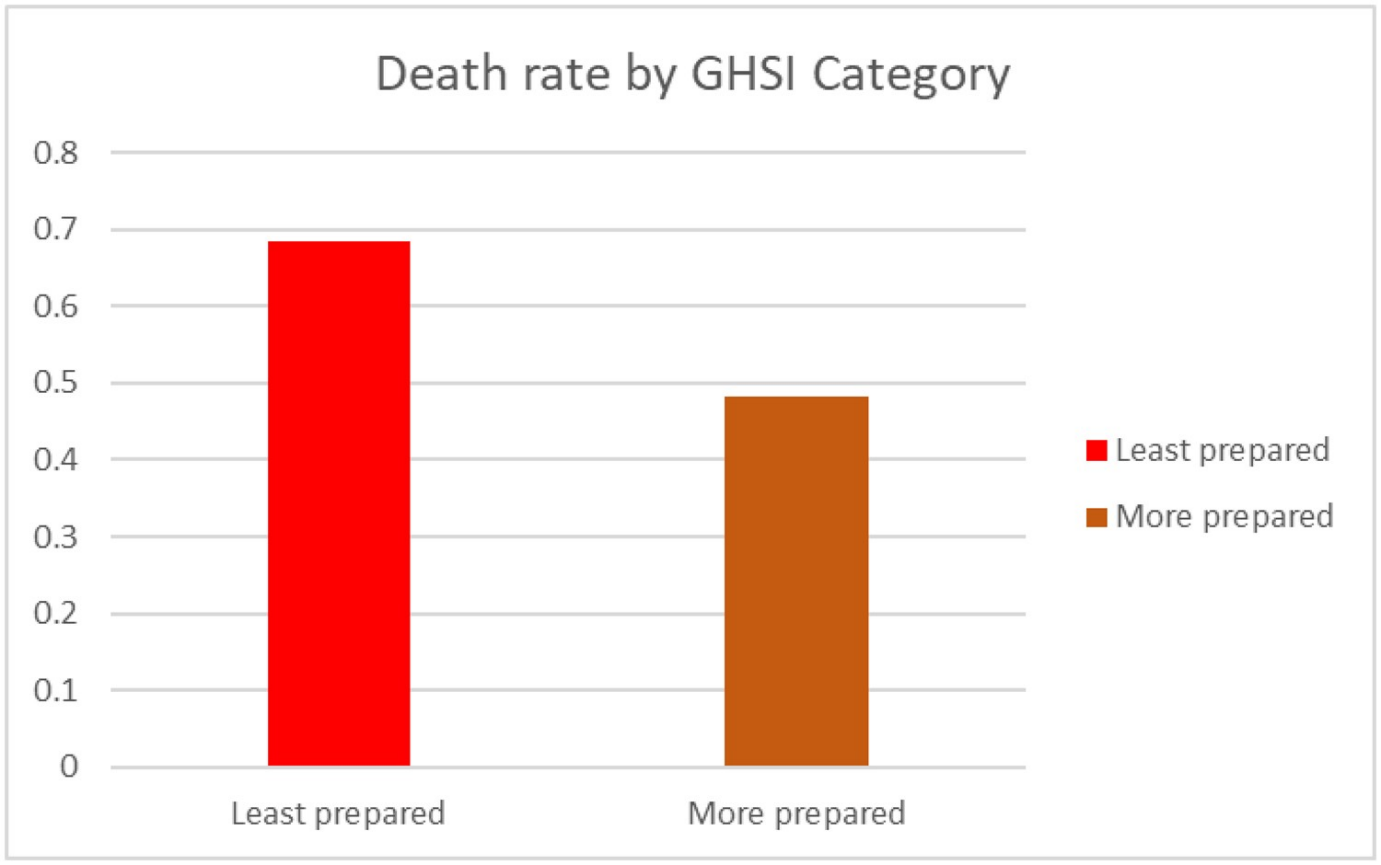
Death rate by GHSI category.

**Fig 9:**
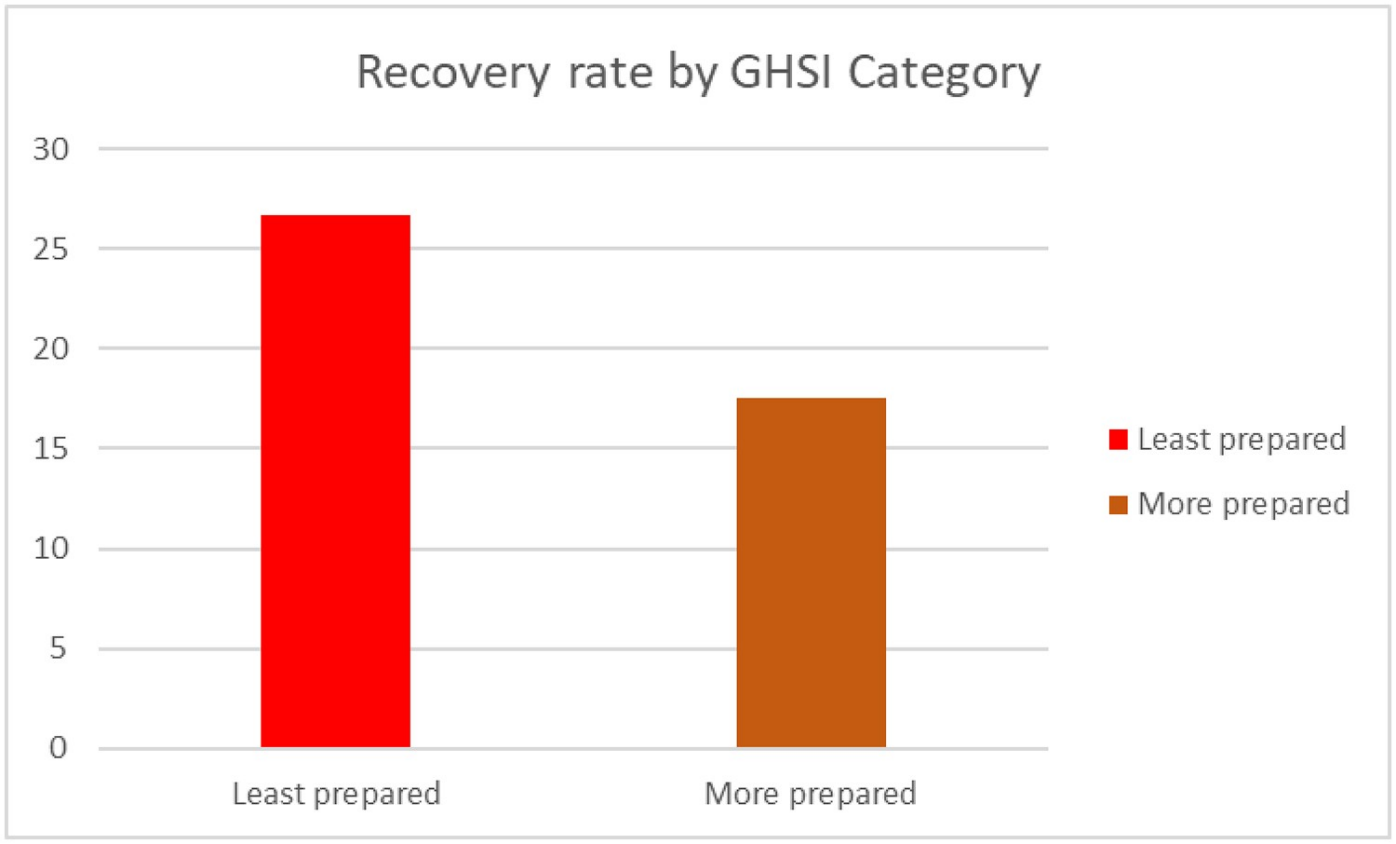
Recovery rate by GHSI category.

Also, the death rate (number of deaths (per million)/confirmed positive cases (per million)) was highest in the least prepared countries (see Fig 8). Strikingly, the recovery rate (number of recoveries (per million)/confirmed positive cases (per million)) was highest in the least prepared countries (Fig 9).

Spearman’s rho correlation coefficients among selected variables revealed varied effect sizes from weak to strong, as well as significant associations (Table 1). Strong statistically significant correlations (0.5 and above) are in boldface from Table 1. From Table 1, total deaths (per million) show strong positive correlations with total cases (per million); median age; aged 65 older; aged 70 older; GDP per capita; hospital beds per thousand; Human Development Index (HDI); recoveries (per million); and overall risk environment with *r*_*s*_ above 0.5, and weak correlations (*r*_*s*_ <0.5) with life expectancy; handwashing facilities; and male smokers). Negative correlations exist between deaths (per million) and extreme poverty, and commitments. The total number of COVID-19 cases (per million) has strong positive correlations (*r*_*s*_ >0.5) with the median age; aged 65 older; aged 70 older; GDP per capita; number of hospital beds per thousand; HDI; recoveries (per million); and the overall risk environment of country, and weak correlations handwashing facility (*r*_*s*_ = 0.392) and life expectancy (*r*_*s*_ = 0.447). It, however, shows negative correlations with extreme poverty (*r*_*s*_ = -0.636) and commitments (*r*_*s*_ = -0.303). The total recoveries (per million) showed strong positive correlations (*r*_*s*_ >0.5) with the total number of positive cases; total deaths (per million); median age; aged 70 older; GDP per capita; the number of hospital bed per thousand; and HDI. Weak positive correlations (*r*_*s*_ <0.5) existed between total recoveries (per million) and aged 65 older and handwashing facilities, and life expectancy. There were strong negative correlations between the total number of recoveries (per million) and population density and extreme poverty.

**Table 1:** Spearman’s rank-order correlations between variables.

**Variable**	**Total cases permillion**	**Total deaths per million**	**Population density**	**Median age**	**Aged 65 older**	**Aged 70 older**	**GDP per capita**	**Extreme poverty**	**Cardiovasc death rate**	**Diabetes prevalence**	**Female smokers**	**Male smokers**
Total cases permillion	1.000											
Total deaths per million	.870**	1.000										
Population density	-.094	-.167	1.000									
Median age	.715**	.668**	.059	1.000								
Aged 65 older	.538**	.501**	-.101	.790**	1.000							
Aged 70 older	.596**	.591**	-.093	.773**	.952**	1.000						
GDP per capita	.645**	.585**	-.137	.656**	.517**	.566**	1.000					
Extreme poverty	-.636**	-.590**	-.002	-.644**	-.551**	-.611**	-.740**	1.000				
Cardiovasc death rate	.025	.124	-.256	-.024	.112	.064	-.178	.045	1.000			
Diabetes prevalence	.139	.157	-.069	.452**	.529**	.502**	.342*	-.381*	.146	1.000		
Female smokers	.164	-.112	.000	.134	-.008	.000	.112	.163	-.262	.073	1.000	
Male smokers	.365*	.366*	-.220	.403*	.401*	.431**	.412*	-.315	.436**	.340*	.369*	1.000
Handwashing facilities	.392**	.340*	-.159	.322*	.213	.317*	.629**	-.475**	.024	.109	.056	.317
Hospital beds per thousand	.701**	.701**	.064	.666**	.494**	.602**	.685**	-.623**	-.131	.422**	.451*	.567**
Life expectancy	.447**	.413**	.139	.599**	.521**	.592**	.442**	-.541**	-.204	.296*	.017	.249
HDI	.661**	.572**	.026	.707**	.529**	.611**	.879**	-.639**	-.199	.347*	.183	.434**
Recoveries per million	.977**	.846**	-.054	.699**	.494**	.554**	.637**	-.645**	-.022	.078	.163	.323
Overall GHSI Score	.038	-.024	.337*	.115	-.009	-.048	.034	-.175	-.198	-.216	-.029	.038
Prevention	.011	-.027	.232	.102	.085	.041	.079	-.087	-.107	.024	-.046	-.072
Early Detection & Reporting	-.043	-.154	.181	.014	-.145	-.217	-.025	-.168	-.120	-.260	.076	.013
Rapid Response	-.066	-.009	.284*	.030	.015	-.024	-.098	-.033	-.047	-.067	-.106	.059
Sufficient and Rubust HS	.174	.151	.225	.218	.162	.130	.028	-.203	-.165	-.050	-.093	.140
Commitment	-.303*	-.314*	.192	-.317*	-.290*	-.343*	-.308*	.302	-.196	-.294*	-.002	-.266
Overall Risk Environment	.619**	.536**	.216	.627**	.478**	.557**	.727**	-.633**	-.278*	.063	.186	.452**
**Variable**	**Handwashing facilities**	**Hospital beds per thousand**	**Life expectancy**	**HDI**	**Recoveries per million**	**Overall GHSI Score**	**Prevention**	**Early Detection & Reporting**	**Rapid Response**	**Sufficient & Rubust HS**	**Commitment**	**Overall Risk Environment**
Total cases permillion												
Total deaths per million												
Population density												
Median age												
Aged 65 older												
Aged 70 older												
GDP per capita												
Extreme poverty												
Cardiovasc death rate												
Diabetes prevalence												
Female smokers												
Male smokers												
Handwashing facilities	1.000											
Hospital beds per thousand	.275	1.000										
Life expectancy	.389**	.432**	1.000									
HDI	.624**	.751**	.620**	1.000								
Recoveries per million	.355*	.716**	.446**	.664**	1.000							
Overall GHSI Score	.211	-.215	.149	.161	.073	1.000						
Prevention	.296	-.198	.189	.183	-.007	.716**	1.000					
Early Detection & Reporting	.138	-.239	-.009	.055	.009	.844**	.566**	1.000				
Rapid Response	.140	-.231	.055	-.029	-.089	.758**	.508**	.486**	1.000			
Sufficient and Rubust HS	-.034	.047	.227	.132	.167	.636**	.499**	.423**	.476**	1.000		
Commitment	-.015	-.458**	-.201	-.228	-.255	.567**	.248	.405**	.422**	.173	1.000	
Overall Risk Environment	.453**	.635**	.596**	.794**	.635**	.292*	.191	.102	.130	.260	-.165	1.000

** Significant correlation at p<0.0.1.

* Significant correlation at p<0.05.

### Multivariable analyses results

#### Linear regression of total confirmed positive cases (per million) and predictor variables

Table 2 shows the coefficient (coef.), robust standard error (SE), probability value (P-value) and the confidence intervals (conf. interval) associated with the predictors of the total confirmed COVID-19 positive cases (per million) among countries. From the model output (Table 2), Male smokers and recoveries (per million) revealed a direct (positive) significant association with a change in the total confirmed positive cases (per million) (p<0.05). Similarly, change in handwashing facilities, hospital beds per thousand, and the overall risk environment showed inversely significant association with a change in the total confirmed positive cases (per million) (p<0.05).

**Table 2:** Linear regression (multivariable) of total cases per million and predictor variables.

Variable (R-squared = 0.998; p<0.001)	Coeff.	SE	P value	95% Conf. Interval	
Total deaths per million	-0.380	6.853	0.062	-30.072	0.931
Median age	0.163	68.015	0.164	-50.695	257.026
Aged 65 and older	-0.269	424.893	0.250	-1483.069	439.283
Aged 70 and older	0.242	569.849	0.225	-545.892	2032.283
Male smokers	0.208	12.772	0.007	15.352	73.136
Handwashing facilities	-0.128	4.509	0.015	-23.722	-3.321
Hospital beds per thousand	-0.164	203.904	0.013	-1089.111	-166.583
Life expectancy	0.065	33.625	0.282	-37.550	114.579
HDI	0.133	1936.720	0.109	-931.505	7830.825
Recoveries per million	1.381	208328.774	0.000	1072546.637	2015091.493
Commitment	-0.006	6.427	0.814	-16.092	12.985
Overall Risk Environment	-0.142	16.755	0.023	-83.867	-8.064

The R-squared of the model output was 0.998 (P<0.001). This implies that the predictor variables had a 99.8% predictive power of the variance in the dependent variable (total confirmed positive cases (per million).

#### Linear regression of total cases of deaths (per million) and predictor variables

Table 3 shows the coefficient (coef.), robust standard error (SE), probability value (P-value) and the confidence intervals (conf. interval) associated with the predictors of the total number of COVID-19 death cases (Deaths) among countries. The model output showed that two predictors (male smokers and recoveries (per million)) significantly associated with the total cases of deaths (per million) (P<0.05) directly.

**Table 3:** Linear regression (multivariable) of total cases of deaths per million and predictor variables.

Variable (R-squared = 0.996; p<0.001)	Coeff.	SE	P value	95% Conf. Interval	
Median age	-0.028	3.395	0.897	-8.766	7.847
Aged 65 older	0.120	19.068	0.762	-40.614	52.701
Aged 70 older	-0.073	23.213	0.812	-62.570	51.031
GDP per capita	0.307	0.002	0.066	0.000	0.011
Extreme poverty	-0.035	0.185	0.535	-0.575	0.331
Male smokers	0.231	0.337	0.009	0.444	2.092
Handwashing facilities	-0.163	0.273	0.152	-1.117	0.220
Hospital beds per thousand	-0.057	7.276	0.433	-23.921	11.685
Life expectancy	-0.064	1.692	0.567	-5.167	3.116
HDI	0.025	94.217	0.862	-213.468	247.616
Recoveries per million	0.836	2406.699	0.000	19385.321	31163.280
Commitment	0.076	0.298	0.149	-0.236	1.223
Overall Risk Environment	-0.072	0.660	0.376	-2.246	0.983

The predictors accounted for 99.6% of the variance in the total cases of deaths (per million) as the model gave an R-squared value of 0.996 (P<0.001).

#### Linear regression of COVID-19 total recoveries (per million) cases and predictor variables

The multivariate model (Table 4) revealed only a positive significant association between total confirmed COVID-19 positive cases (per million) (P<0.001) and COVID-19 total recoveries (per million) cases.

**Table 4:** Linear regression (multivariable) of total recoveries per million and predictor variables.

Variable (R-squared = 0.994; p<0.001)	Coeff.	SE	P value	95% Conf. Interval	
Total cases per million	1.050	0.118	0.000	0.680	1.188
Total deaths per million	-0.028	4.936	0.853	-11.599	9.729
Median age	-0.022	64.276	0.852	-151.109	126.612
Aged 65 older	-0.062	457.208	0.820	-1093.907	881.568
Aged 70 older	-0.165	591.016	0.468	-1718.964	834.660
GDP per capita	0.176	0.055	0.076	-0.013	0.223
Extreme poverty	0.007	3.050	0.824	-5.898	7.281
Handwashing facilities	0.060	4.530	0.256	-4.399	15.174
Hospital beds per thousand	0.038	128.640	0.392	-163.948	391.870
Life expectancy	-0.046	24.704	0.367	-76.456	30.282
HDI	-0.065	1675.871	0.401	-5077.018	2163.979

The R-squared value (0.994) obtained for the model (P<0.001) signified that about 99.4% of the variance in the total COVID-19 recoveries (per million) cases, is accounted for by the predictor variables in the regression model.

## Discussion

The current study sought to assess the strength of health systems of selected African countries via their response capacities (e.g., prevention, early detection and reporting, rapid response) to the on-going COVID-19 outbreak as a function of their confirmed cases, case fatality and recovery cases using the GHSI metrics.

Overall, 54 African countries with COVID-19 cases were included in the study. Preliminary findings show that countries from Western Africa recorded the highest number of confirmed COVID-19 positive cases (26, 679 per million), with the lowest figures being recorded in countries from Eastern Africa (14,421 per million). For COVID-19 related deaths, the highest and least numbers were noted in Southern Africa (514 per million) and Eastern Africa (185 per million) respectively. The number of recoveries was highest in Southern Africa (4,709 per million) whiles Eastern Africa showed the least number of recoveries (717 per million). However, the highest COVID-19 death rate occurred in Northern Africa (0.029), with Eastern Africa recording the lowest death rate (0.015) (see Figs 3 and 4). As expected, the mortality rate (i.e., total deaths (per million)) was shown to be proportional to the total confirmed cases (per million) from the correlation results. Spatial or geographical heterogeneity are vital drivers of disease onset, transmissions, deaths and recoveries, suggesting that certain socioeconomic features might be related to disease pattern in one region at the expense of the other owing to regional variations in culture or behavioural norms, transportation systems and/ or the patterns of human movement to explain the dynamics in disease spread [25, 26]. For example, sub-regional countries (e.g., Southern- South Africa; Northern- Egypt, Morocco; Western- Ghana, Nigeria; Eastern- Kenya, Ethiopia) with more socially open economies for more international arrivals might have acted as a proxy for the spread of the COVID-19 disease right from the onset of the virus outbreak. Again, countries (e.g., Egypt, South Africa) with more advanced transport infrastructure might have facilitated the widespread of the disease because the mobility of persons within and between cities is quite easier [27]. Hence, higher mobility increases the spread of the disease, with more persons infected more likely to observe a considerable number of deaths. Similarly, the economic capacity of most African countries varies markedly. Therefore, countries like Algeria, Egypt and South Africa (categorized as high-income and upper-middle-income economies) might be well equipped and better prepared to face the pandemic than countries, for example, Tunisia, Morocco or Ghana (classified lower-middle-income countries). Hence, the availability of health logistics and facilities for case classification as well as personnel might be adequate in some countries and low in others of the region.

Current findings also show that patient-level characteristics such as ageing population (i.e., 65^+^), poverty, underlying co-morbidities–cardiovascular disease (e.g., hypertension), and diabetes through health-compromising behaviours like smoking as well as hospital care (i.e., beds per thousand) can help explain COVID-19 confirmed cases and mortality rates in Africa [28–32]. These findings could provide useful information for health professionals to identify high-risk COVID-19 patients. However, this evidence alone might not be enough to offer effective policies for reducing COVID-19 mortality on the continent [29]. These findings emphasize the essential role of public awareness about hand hygiene (i.e., hand washing) as a public health prevention strategy towards the on-going pandemic [33].

Except for age, the GHS index parameters (e.g., population, prevention, detection and reporting, rapid response) were significant and positively correlated with the prevalence, mortality and recoveries of COVID-19 in the African region. For instance, the degree of an epidemic like COVID-19 spread inversely with population density and structured populations [34]. This trend implies that all things being equal, in densely populated regions, it is quite simple to detect and aim at persons who are more susceptible to a disease condition with planned interventions [35]. Usually, these are the individuals who are more socially connected so are easily and early detected with a disease condition [36]. The number of confirmed cases corresponded with the number of recovered cases and deaths but with various rapid response strategies across different regions of the continent as a caveat. Similar to the national health statistics, Africa’s demographic structure may influence the susceptibility of the population to a disease condition like COVID-19. Age was an inverse determinant of the number of COVID-19 deaths and recoveries. Some age groups (65+, older adults) usually have weaker defensive health mechanisms because of ageing-related physical and physiological degeneration to cope with disease conditions (i.e., multi-morbidities) compared to children (i.e., <15 years) with greater defensive mechanisms [37–40]. This finding corroborates a finding from the Center for Disease Control and Prevention in the United States. Using data up to May 20, 2020, 81% and 94% of COVID-19 deaths were persons aged 65 and 55 and over in the US respectively [41]. Similarly, (32) assessed fatality risk in the province of Wuhan in China and revealed that, compared to 30-59year olds, those under 30 years of age were 0.6 times more likely and those over 60 years of age were 5.1 times more likely to die if symptomatic. The surprising correlational outcomes in this study except age might due to other likelihoods such as limited COVID-19 case classification, less testing capacities, inaccurate reporting, Africa’s younger populations, and differences in climate and humidity [42].

Consistent with previous studies [43–46], other results from this study showed that change in overall GHSI, prevention, detection and reporting, rapid response, sufficient and robust HS and overall risk environment was significantly associated with a change in the confirmed positive cases and deaths. Therefore, the general risky environment and country’s susceptibility to COVID-19 threats might be connected to the degree of cumulative incidence, as the rate of incidence and mortality of COVID-19 increase. The implication is that countries with higher GHSI metrics (e.g., better prepared with prevention strategies, early detection, rapid response), all things being equal, were more likely to have less increase in prevalence and mortality rates with corresponding better recoveries [47]. For example [46], revealed that the application of three non-pharmaceutical interventions; school closure, banning of public meetings, and isolation, as well as quarantine on the influenza pandemic in the United States between 1918 to 1919, was significantly linked with less weekly additional death rates, more intervals in getting peak mortality, and lower total deaths, especially when these interventions were early employed over a considerable period.

Although the high correlations were observed between the overall GHSI metrics (i.e., prevention, early detection & reporting, rapid response, sufficient & robust), a low correlation was realized between overall GHSI and overall risk environment for COVID-19 in the African region. This pattern observed demonstrates the lack of utility of the GHS index in predicting the response of countries to the COVID-19 pandemic and its impact on the noted COVID-19 performance indicators. Similar GHSI findings have been reported in similar studies across developed countries [48]. Specifically, though Africa was classified as one of the unprepared regions of the world using the GHS index as a benchmark; the overall continent has been relatively successful with control of the ongoing pandemic. The foregoing highlights the key role leadership plays in crisis management. Decisive leadership and coordinated responses by African leaders and supportive agencies (e.g., Africa Centre for Disease Control, international partners- WHO) have been pivotal towards country-specific rapid response (e.g., mobilizing critical resources) to the on-going pandemic. Although the GHSI metrics could be a useful measure in predicting the preparedness of countries toward outbreaks such as the current pandemic, identified ratings could either overestimate or underestimate the rigorousness of some national health care systems and their level of preparedness [48].

Some other parameters (e.g., life expectancy, death rate, health expenditure) also influence the likelihood of recoveries and deaths after COVID-19 infection [27]. According to Valero and partner, for developed (i.e., high-income) countries, more deaths occur because of higher life expectancy. For instance, in China [49], revealed that the COVID-19 case fatality rate was 3.67%. This figure reduced (i.e., averaged 1.38%) when demography and under-ascertainment were adjusted but still found higher rates for older age groups: 0.32% in those aged <60 years vs. 6.4% in those aged >60 years, up to 13.4% in those aged 80 years or older. High life expectancy is often related to an older population, and death data implies that COVID-19 is more predominant for such population compared to Africa with the youngest population of the world [50], though the strength of its health system might play a critical role in the determination of the number of deaths and recoveries. Therefore, an effective health system (e.g., better hospitals facilities—ICUs, testing laboratories, personal protective equipment, properly trained health personnel) in Africa could help better identify COVID-related deaths and also facilitate more recoveries [51]. Hence, countries with more effective health systems might experience less COVID-19 related deaths. Low-to-middle income countries will seriously suffer from inefficient health systems.

### Strengths and limitations

To the best of authors’ knowledge, the current study is the first on the African continent to show higher global health security metrics as a function of better preparedness and related capacities in the sub-regional states and its implication on how to mitigate and control the COVID-19 pandemic. Measuring the GHS index for Africa may help in improving timeliness in the detection and response to future disease outbreaks as well as increase the current health security at the sub-regional levels because of COVID-19. We infer that countries committed to the virus may work towards the GHS metrics. The strong positive association between GHS index and prevalence and/ or mortality of COVID-19 might help prioritize the country’s healthcare system to facilitate real-life changes in incidence/mortality cases of the current pandemic and potential outbreaks.

Despite these strengths, the current study is not without limitations. Like any ecological study, some ecological inconsistencies in the current work may exist because of overlapping national-level data including total deaths, testing done, and the recoveries among others. The cross-sectional nature of the design means that it is difficult to draw any causal conclusions to the obtained findings. Also, the variability in COVID-19 reporting concerning testing capacities at the country level and not accounting for time-dependent reporting of cases as well as time-lag in outcomes might cause underestimation of the exact number of cases and deaths from COVID-19547 in some parts of the African region. Though the GHS index provides clear and precise descriptions of the parameters, finding the appropriate data points remains contentious. It is unclear whether processes such as quicker detection of COVID-19 cases will reduce the total number of cases in the country as measured by the GHS index and help improve health outcomes in a population.

### Practical implications

Countries with high capacities based on the benchmarks provided by the GHSI metrics are more likely to manage COVID-19 related cases (i.e., prevalence, deaths and recoveries) better, although with caution. Findings of the current study have shown that pandemic preparedness hinges on a country’s capacity to early detect & report, sufficient & robust health system, commitments to improve national capacity, and its overall risk environment. The call is on national governments to design and implement appropriate interventions that would help fight the on-going pandemic and potential infectious disease outbreaks in the future using standard health benchmarks such as GHS index as a framework for countries where their health security capacities require upgrade.

## Conclusion

The study assessed the strength of health systems of selected African countries through their response capacities (e.g., prevention, early detection and reporting, rapid response) to the on-going COVID-19 outbreak as a function of its confirmed cases, case fatality and recoveries using the Global Health Security Index (GHSI). Findings suggest that patient-level characteristics such as ageing population (i.e., 65^+^), poverty, underlying co-morbidities–cardiovascular disease (e.g., hypertension), and diabetes through health-compromising behaviours like smoking as well as hospital care (i.e., beds per thousand) can help explain COVID-19 confirmed cases and mortality rates in Africa. Other determinants (e.g., population density, the ability of detection, prevention and control) also affect COVID-19 prevalence, deaths and recoveries within African countries and sub-regions. Despite the no and low correlations noted on few indices of the GHS index in the current and previous studies, it still serves as a useful framework that can positively impact the development of appropriate interventions that offer stronger guarantees for health security concerning the current pandemic and perhaps future infectious outbreaks. The pattern of the virus on the continent requires improvements in health capacity building and collaboration between countries for optimal improvement in infectious disease management. Future studies should not only explain sub-regional variations in COVID-19 responses using the GHSI metrics but also variations in the timing, sequencing and speed of adoption of the parameters on the GHSI might help test its robustness.
